# Supplementary material for: The Immunoscore in Localized Urothelial Carcinoma Treated with Neoadjuvant Chemotherapy: Clinical Significance for Pathologic Responses and Overall Survival
Source: Cancers (Basel). 2021 Jan 28;13(3):494. doi: 10.3390/cancers13030494 (PMC7865364; doi:10.3390/cancers13030494)
Supplement: Supplementary file 1 [file cancers-13-00494-s001.pdf]

cancers

MDPI

Supplementary Materials:

The Immunoscore in Localized Urothelial Carcinoma Treated with Neoadjuvant Chemotherapy: Clinical Significance for Pathologic Responses and Overall Survival

Elise F. Nassif, Bernhard Mlecnik, Constance Thibault, Marie Auvray, Daniela Bruni, Alexandre Colau, Eva Compérat, Gabriela Bindea, Aurélie Catteau, Aurélie Fugon, Isabelle Boquet, Marine Martel, Philippe Camparo, Pierre Colin, Roubini Zakopoulou, Aristotelis Bamias, Mostefa Bennamoun, Xavier Barthere, Bruno D'acremont, Marine Lefevre, Francois Audenet, Arnaud Mejean, Virginie Verkarre, Stéphane Oudard and Jérôme Galon

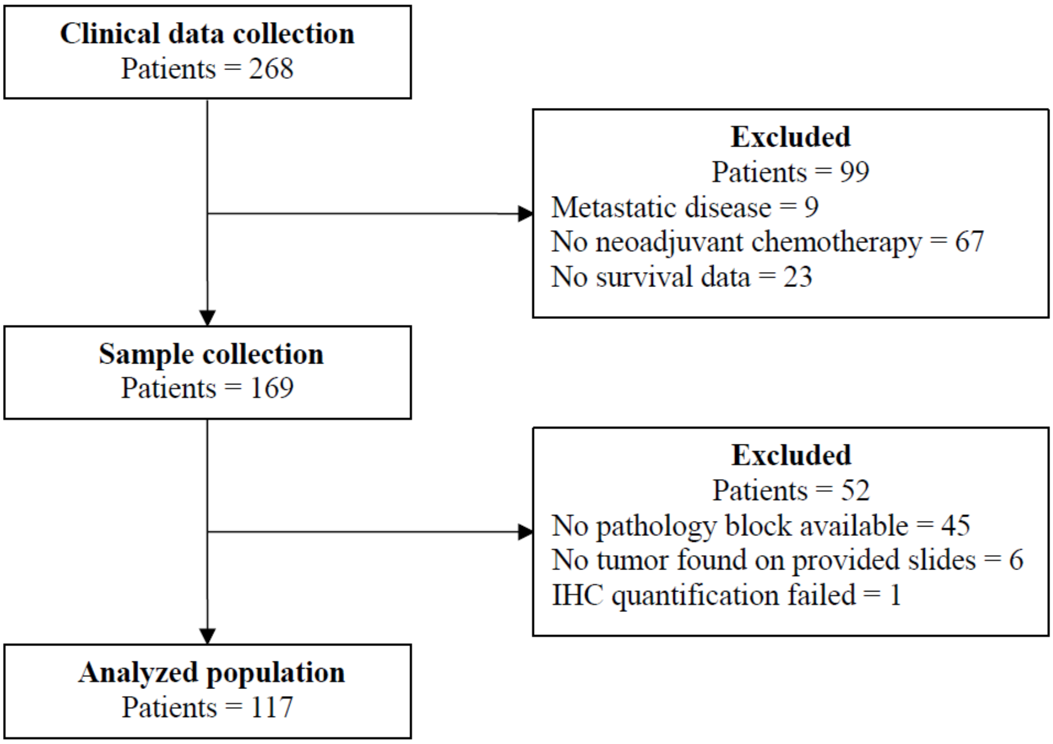

Figure S1. The localized UC study design. Biomarker exclusion and clinical a data exclusion prior to analysis.

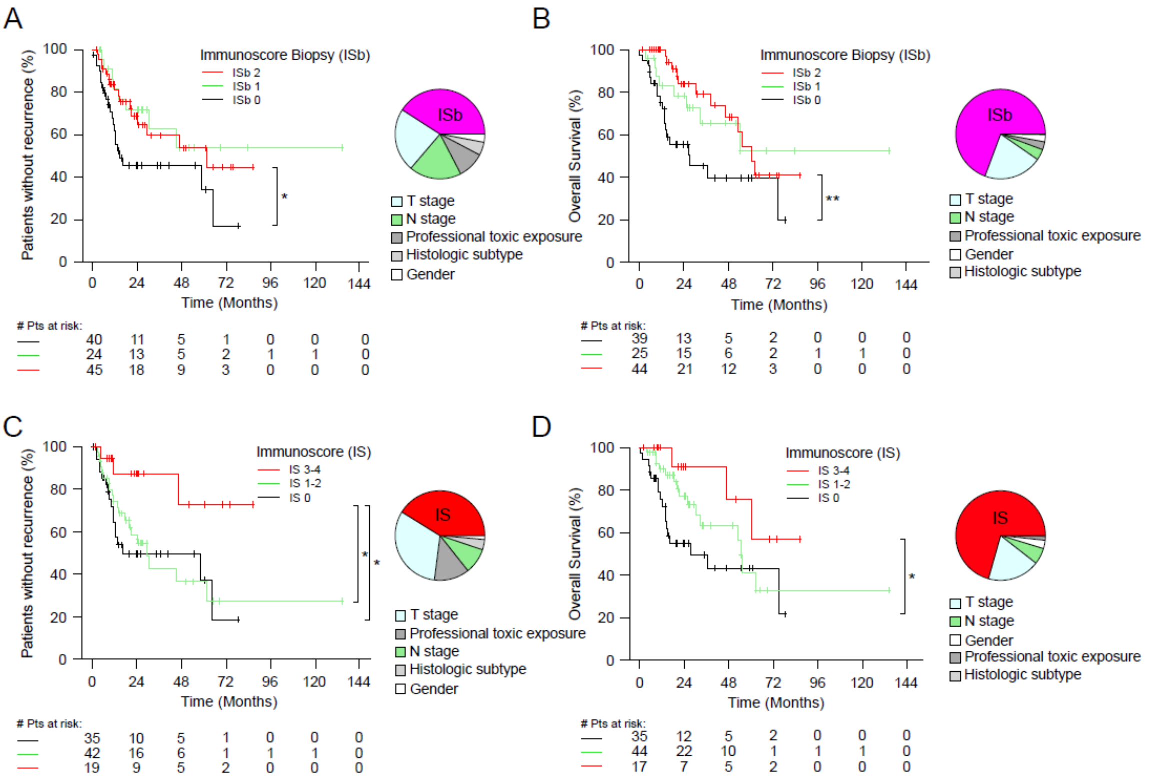

Figure S2. The impact of Immunoscore on the patient outcome.Ð'dKaplan-Meier curves for Immunoscore (IS) are shown for TTR (A, C) and OS (B, D). Pie chart indicates the contribution of X2 proportion of ISb and IS among other variables for influencing survival in multivariate analysis. (A-B) Immunoscore three categories for Immunoscore biopsy: ISb 0 (black), ISb 1 (green) and ISb 2 (red). (C-D) Immunoscore three categories for Immunoscore: IS 0 (black), IS1-2 (green) and IS 3-4 (red). * P<0.05; ** P < 0.01.

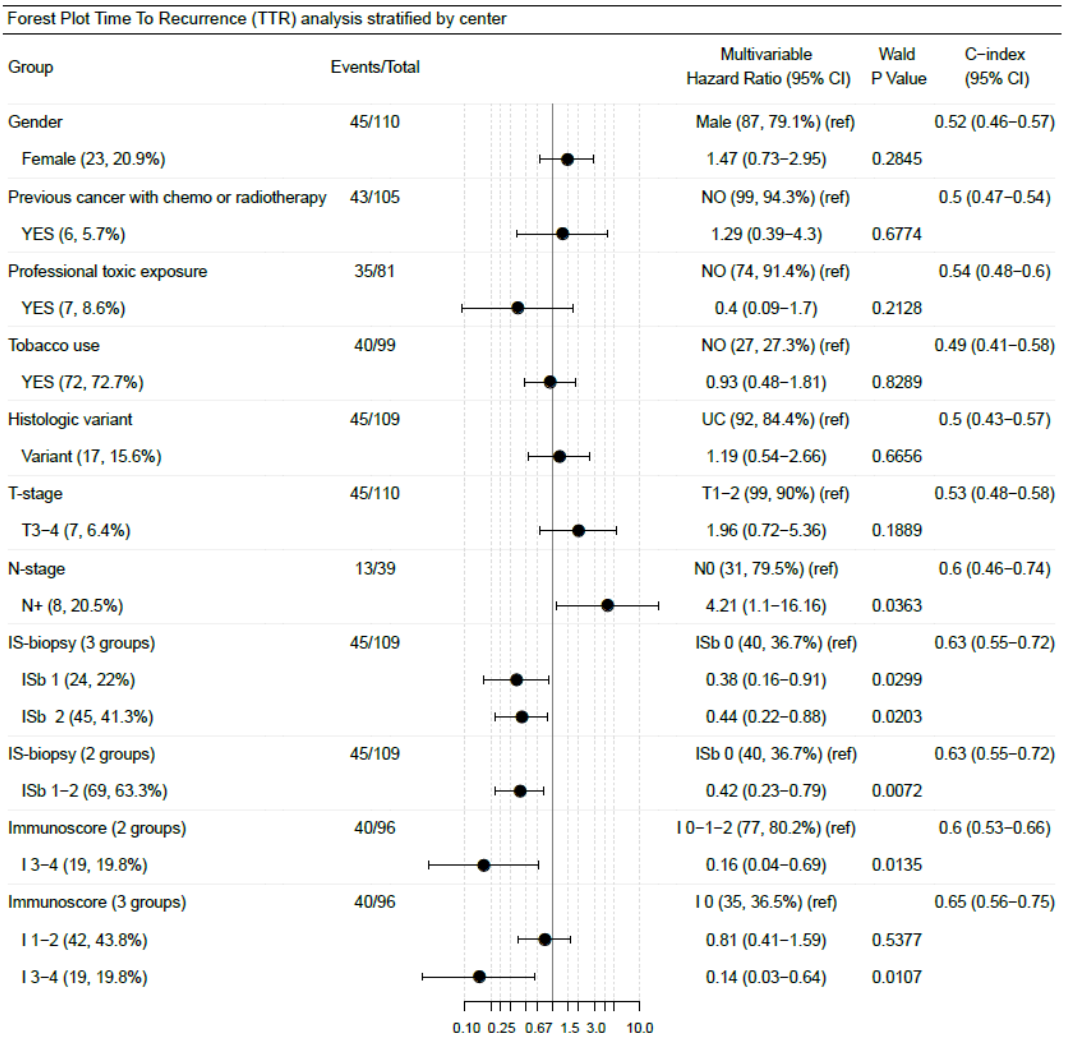

Figure S3. Forest plot for the impact of clinical parameters and Immunoscore on the patient outcome.Đ'dBi-variate Cox models stratified by participating center for clinical parameters and Immunoscore.

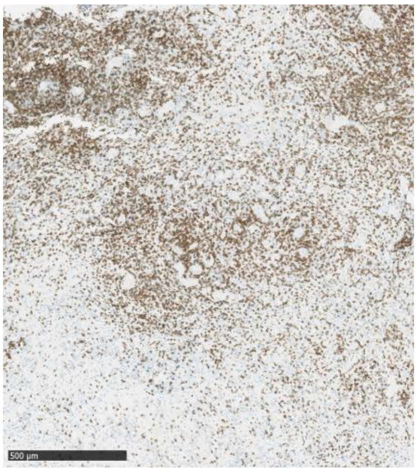

A

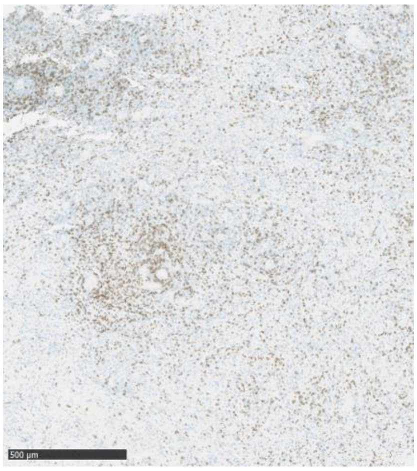

B

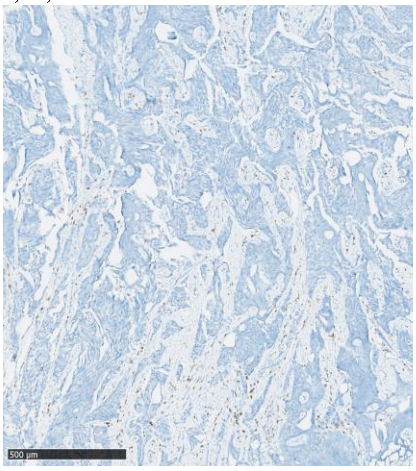
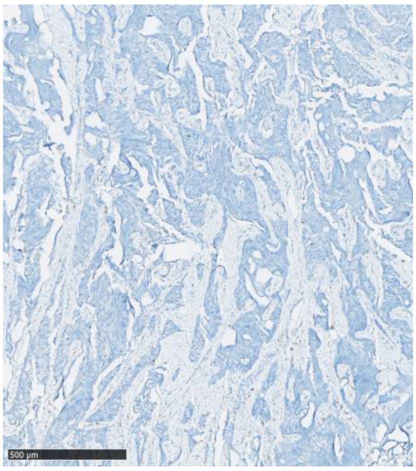

C D

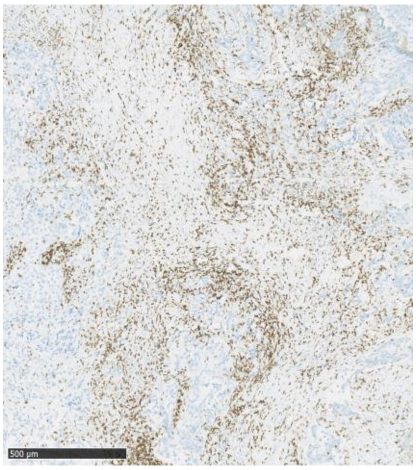
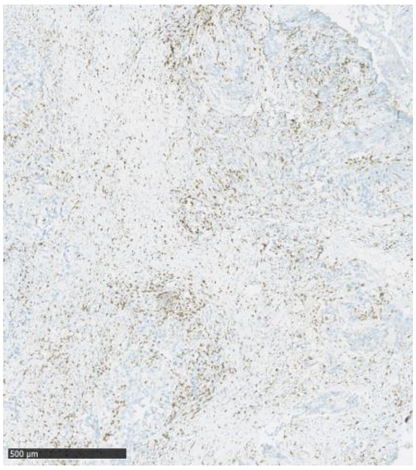

E F

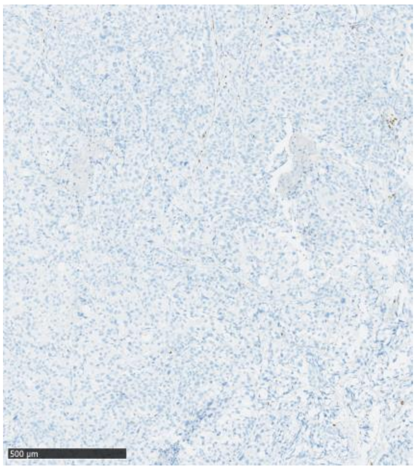
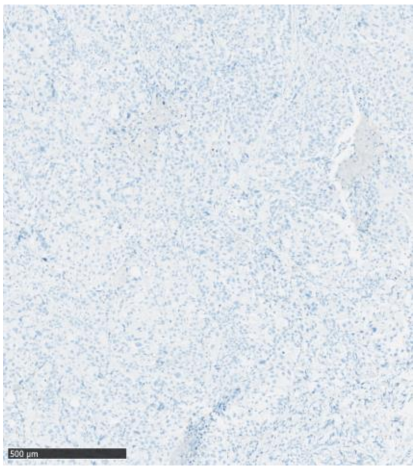

G H

Figure S4. Pathology slides for Immunoscore determinationĐA) CD3 immunohistochemistry (IHC) in IS-4 patient (B) CD8 IHC in IS-4 patient (C) CD3 IHC in IS-0 patient (D) CD8 IHC in IS-0 patient (E) CD3 IHC in ISb-2 patient (F) CD8 IHC in ISb-2 patient (G) CD3.

B

Table S1: Bivariable analysis for Clinical Parameters for OS

	Number of patients (%)	Overall Survival (OS): Median months (95% CI)	Rate at 3 yr % (95% CI)	Rate at 5 yr % (95% CI)	Unadjusted stratified by center: HR (95% CI)	P value*	C-index (95% CI)	RMST Rel. Months (95% CI)	P value**
**Sex**							0.51 (0.43−0.58)		
Male	86 (78.9)	60.5 (32.9-NR)	62.7 (51.5-76.4)	50.2 (37.3-67.5)	1.0 (reference)			0.0 (reference)	
Female	23 (21.1)	53.1 (5.9-74.8)	64.3 (44.5-92.8)	37.5 (15-94)	0.99 (0.44−2.22)	0,9828		-2.3 (-17.9-13.2)	0,7683
**Previous cancer with chemotherapy or pelvic radiotherapy**							0.5 (0.45−0.54)		
No	100 (95.2)	55.3 (32.9-74.8)	64.1 (53.8-76.3)	48.1 (35.8-64.5)	1.0 (reference)			0.0 (reference)	
Yes	5 (4.8)	NR	80 (51.6-100)	NR (NR-NR)	0.76 (0.1−5.72)	0,7914		6.5 (-9.4-22.4)	0,4242
**Professional toxic exposure**							0.52 (0.43−0.6)		
No	73 (89)	55.3 (27.4-NR)	60.7 (48.6-75.7)	46.3 (32.5-65.9)	1.0 (reference)			0.0 (reference)	
Yes	9 (11)	NR	77.8 (54.9-100)	77.8 (54.9-100)	0.64 (0.19−2.16)	0,4688		9.8 (-13.1-32.7)	0,4001
**Tobacco use**							0.54 (0.44−0.63)		
No	24 (24.7)	74.8 (5.2-NR)	63.9 (45.2-90.3)	63.9 (45.2-90.3)	1.0 (reference)			0.0 (reference)	
Yes	73 (75.3)	55.3 (30.6-NR)	63 (50.8-78)	46.3 (31.7-67.6)	1.51 (0.67−3.44)	0,3220		-8.4 (-25-8.2)	0,3202
**Histologic variant**							0.51 (0.43−0.58)		
UC	93 (85.3)	55.3 (32.9-NR)	64.9 (54.1-77.8)	47.7 (34.2-66.6)	1.0 (reference)			0.0 (reference)	
Variant	16 (14.7)	36.8 (12.4-74.8)	53.4 (31.3-91.2)	42.7 (21.4-85.3)	1.4 (0.64−3.08)	0,3990		-7.8 (-24.5-8.8)	0,3557
**T-stage**							0.49 (0.47−0.51)		
T2	99 (90.8)	60.5 (36.8-NR)	66.2 (56.2-78)	52.4 (40.2-68.4)	1.0 (reference)			0.0 (reference)	
T3-4	6 (5.5)	25.8 (17.5-30.6)	NR (NR-NR)	NR (NR-NR)	1.85 (0.52−6.58)	0,3421		1.3 (-3.7-6.3)	0,6124
**N-stage**							0.55 (0.38−0.73)		
N0	26 (76.5)	36.8 (0.1-NR)	74.6 (53-100)	37.3 (8.9-100)	1.0 (reference)			0.0 (reference)	
N+	8 (23.5)	25.8 (9.9-NR)	41.7 (14.7-100)	NR (NR-NR)	1.96 (0.31−12.36)	0,4734		-4.7 (-20.4-11)	0,5575
**IS-biopsy (2 groups)**							0.7 (0.61−0.78)		
ISb 0	39 (36.1)	27.4 (13.9-74.8)	45.4 (29.9-68.8)	39.7 (24.3-64.9)	1.0 (reference)			0.0 (reference)	
ISb 1-2	69 (63.9)	60.5 (38.8-NR)	73.8 (62-87.8)	53.2 (37.8-74.8)	0.3 (0.15−0.59)	0,0005		15.8 (2.1-29.6)	0,0244
**IS-biopsy (3 groups)**							0.73 (0.65−0.8)		
ISb 0	39 (36.1)	27.4 (13.9-74.8)	45.4 (29.9-68.8)	39.7 (24.3-64.9)	1.0 (reference)			0.0 (reference)	
ISb 1	25 (23.1)	NR	65.4 (46.9-91.2)	52.4 (30.2-90.7)	0.34 (0.14−0.82)	0,0167		14.6 (-2.9-32)	0,1018
ISb 2	44 (40.7)	60.5 (38.8-NR)	79.2 (65.3-95.9)	54.6 (35.9-83)	0.27 (0.12−0.6)	0,0013		17.2 (2.5-31.8)	0,0216
**Immunoscore (2 groups)**							0.59 (0.53−0.65)		
IS 0-1-2	79 (82.3)	54.4 (27.4-74.8)	57.3 (45.5-72.3)	41.9 (28.3-62)	1.0 (reference)			0.0 (reference)	
IS 3-4	17 (17.7)	NR	90.9 (75.4-100)	75.8 (50.6-100)	0.35 (0.1−1.19)	0,0936		21.2 (3.3-39.1)	0,0200
**Immunoscore (3 groups)**							0.71 (0.62−0.79)		
IS 0	35 (36.5)	27.4 (12.4-74.8)	49.5 (33.7-72.6)	43.3 (27.2-68.9)	1.0 (reference)			0.0 (reference)	
IS 1-2	44 (45.8)	54.4 (21.2-NR)	63.4 (47.5-84.7)	41.1 (23.1-73.3)	0.43 (0.2−0.91)	0,0281		8.9 (-6.7-24.4)	0,2630
IS 3-4	17 (17.7)	NR	90.9 (75.4-100)	75.8 (50.6-100)	0.22 (0.06−0.78)	0,0197		23.1 (5.5-40.7)	0,0101

* Wald P Value stratified by participating center. ** Restricted Mean Survival Time (RMST) P value. IS: Immunoscore; ISb Immunoscore biopsy like.

Table S2: Multivariable analysis Immunoscore vs clinical parameters for TTR and OS

	TTR Model (45/110)*: Hazard Ratio (95% CI)	P-value[1]	C-Index (95% CI)	OS Model (40/110)*: Hazard Ratio (95% CI)	P-value[1]	C-Index (95% CI)
**Multivariable Cox Model stratified by center**			0,71 (0.62-0.79)			0,7 (0.6-0.79)
**Immunoscore (3 groups)**						
IS 1-2 *vs* IS 0	0.62 (0.29-1.33)	0,2214		0.35 (0.16-0.8)	0,0121	
IS 3-4 *vs* IS 0	0.14 (0.03-0.67)	0,0134		0.17 (0.04-0.66)	0,011	
Unclassified *vs* IS 0	0.35 (0.12-1.05)	0,0602		0.37 (0.12-1.17)	0,0908	
**Gender**						
Female *vs* Male	1.23 (0.59-2.56)	0,5828		0.75 (0.32-1.77)	0,5087	
**Professional toxic exposure**						
Yes *vs* No	0.25 (0.04-1.45)	0,1204		0.75 (0.19-2.93)	0,6754	
Unkown *vs* No	0.71 (0.22-2.31)	0,5637		0.88 (0.26-3.02)	0,8451	
**Histologic variant**						
Variant *vs* UC	0.67 (0.26-1.71)	0,4009		1.11 (0.44-2.78)	0,8244	
Unkown *vs* UC	0 (0-Inf)	0,9979		-	-	
**T-stage**						
T3-4 *vs* T2	2.42 (0.76-7.67)	0,1345		3.26 (0.81-13.21)	0,0976	
Unknown *vs* T2	5.87 (1.19-28.92)	0,0298		1 (0.18-5.52)	0,9976	
**N-stage**						
N+ *vs* N0	2.48 (0.64-9.59)	0,1882		1.31 (0.23-7.46)	0,7588	
Unknown *vs* N0	1.26 (0.29-5.55)	0,7603		2.11 (0.43-10.39)	0,3577	

* (Events/Total); [1]Stratified covariate Wald p-value; Stratified by center. IS: Immunoscore
